# Dosimetry audits in Taiwan radiotherapy departments

**DOI:** 10.1259/bjro.20210002

**Published:** 2021-04-15

**Authors:** An-Cheng Shiau, Shih-Ming Hsu, Pei-Yun Huang, Chiu-Ping Chen, Yi-Ting Huang, Ke-Yu Lien, Chin-Ping Chen, Shung-Hwei Fan, Shiu-Chen Jeng, Ho-Hsing Chen, Ji-An Liang

**Affiliations:** ^1^ Department of Radiation Oncology, China Medical University Hospital, Taichung, Taiwan; ^2^ Department of Biomedical Imaging and Radiological Sciences, National Yang-Ming University, Taipei, Taiwan; ^3^ Department of Biomedical Imaging and Radiological Science, China Medical University, Taichung, Taiwan; ^4^ Department of Radiation Oncology, Taoyuan Armed Forces General Hospital, Taoyuan, Taiwan; ^5^ Department of Radiation Oncology, Taipei Medical University-associated Wan-Fang Hospital, Taipei, Taiwan; ^6^ Department of Radiation Protection, Atomic Energy Council, Taipei, Taiwan; ^7^ Department of Radiation Oncology, Taipei Medical University Hospital, Taipei, Taiwan; ^8^ Department of Radiation Oncology, Taichung Veterans General Hospital, Taichung, Taiwan; ^9^ Department of Medicine, China Medical University, Taichung, Taiwan

## Abstract

**Objectives:**

This study examines the practice of the regulation of Standards for Medical Exposure Quality Assurance (SMEQA) in Taiwan based on on-site quality audit for radiation therapy systems from 2016 to 2019.

**Methods:**

81 radiation therapy departments, 141 linacs, 9 γ knife systems, 34 high dose rate brachytherapy systems, 20 Tomotherapys, and 6 Cyberknives were audited yearly. Data collection and analysis for each institute’s documents including QA procedure, ion chamber and electrometer calibration reports, and a questionnaire relating to machine type and staffing, were requested first and reviewed by auditors. On-site SMEQA core item measurements, including beam output, beam profile and energy constancy for external beam therapy systems, and the source strength, positioning, and timer accuracy for brachytherapy systems were audited second. More than 300 photon beams and more than 400 electron beams were measured each year.

**Results:**

There were approximately 8.9 radiotherapy units per million population, and 1.2 medical physicists per unit in Taiwan. For the output measurements, more than 78 and 75% of the photon beams and electron beams, respectively, from linacs were with deviations within ±1.0%. Photon beams have lower beam quality measurement deviations than electron beams. Including in-plane and cross-plane measurements, more than 90 and 85% photon and electron beams, respectively, were with flatness consistency within 1.0%. All audit measurements were within the SMEQA acceptance criteria.

**Conclusions:**

According to SMEQA regulations on-site QA audits were successfully carried out from 2016 to 2019 for all Taiwan radiotherapy units. The measurement results showed high quality machine performance in Taiwan.

**Advances in knowledge:**

Dosimetry audits with directly acquired measurement readings have lower uncertainties; allow immediate feedback, discussion, and adjustment in a timely manner. In addition to regulation system establishment and education and training implementation, the machine quality is closely related to machine maintenance implementation.

## Introduction

The need for dosimetry and geometric accuracy has always been recognized as important in radiotherapy. ICRU recommendations state that the dose delivery to the primary target should have accuracy of at least ±5% of the prescribed value.^1^ Considering the complex process involved in delivering a dose to a target, quality assurance (QA) at each step must be implemented with standards required to deliver the treatment in an accurate and consistent manner. One method for ensuring dosimetry consistency and improved accuracy is the quality audit. However, before the audit is implemented, standards for machine QA including the item, frequency and criteria should be established.

Radiotherapy dosimetry audits have been available for a long time. The IAEA introduced the first postal dosimetry service in 1966. The Radiological Physics Centre (RPC) program, MD Anderson, Houston (now called the IROC-H, the Imaging and Radiation Oncology Core—Houston) was implemented in 1977. The ESTRO program was proposed in 1991. The Quality Assurance Network for radiotherapy (EQUAL) project started in 1998.^2–6^ According to the IAEA Dosimetry Audit Network (DAN), 45 organizations in 39 countries confirmed operating dosimetry audit services for radiotherapy in 2017.^7^

In Taiwan, according to the Ionizing Radiation Protection Act (IRPA), the Standards for Medical Exposure Quality Assurance (SMEQA) regulations were implemented in 2005 by the Atomic Energy Council (AEC).^8^ At that time all medical linacs, Co-60 teletherapy systems and high dose-rate (HDR) brachytherapy units using radioactive material were included. Now all γ knife systems, Cyberknives (Accuray Inc., Sunnyvale, CA), Tomotherapys (Accuray Inc., Sunnyvale CA), X-ray simulators, mammography systems, computed tomography systems (CT), and CT simulators were also included. According to SMEQA regulations, all included devices must establish QA programs approved by the AEC to improve radiological diagnosis and therapy quality and reduce unnecessary radiation exposure to patients. According to the SMEQA, QA programs shall include the QA organization, operating procedures, items checked, frequency, data recording worksheets, and policy when the QA result deviates from the criteria. Yearly on-site inspection is conducted by an AEC officer to check the legality of the personnel and equipment of the ongoing QA program, and whether the QA results meet the tolerance specified by the SMEQA.

The QA frequency, procedures, and tolerance for medical therapy devices in the SMEQA refer to the American Association of Physicists in Medicine (AAPM) TG-40, TG-142, TG-148, and NO. 54 reports,^9–12^ and recommendations from vendor acceptance and commissioning procedures. There are now 36 QA medical linac procedures including dosimetry, mechanical, and safety procedures implemented according to the frequency (daily, monthly or annual) assigned in the SMEQA. The reference-point dosimetry method is requested by the AEC and shall follow the well-established protocol, for example, AAPM TG-21,^13^ TG-51,^14^ or IAEA TRS-398^15^ for linac, and AAPM TG-148^11^ for Tomotherapy, and AAPM TG-43^16^ for brachytherapy system. In Taiwan, all reference-point doses for external beam treatment are traceable to the primary standards. The ion chamber calibration factors, including the *N_x_* (derived from *N_k_*) used in TG-21, and the $N_{D,W}^{60-Co}$ used in TG-51 and TRS-398, were provided directly by the National Radiation Standard Laboratory (NRSL, Taiwan), a primary standards dosimetry laboratory (PSDL), with expanded uncertainty at *k* = 2 about 1%. The ^192^Ir is the source exclusively used for HDR brachytherapy in Taiwan. Well-type chambers with air kerma calibration factors provided by NRSL, with expanded uncertainty about 2.8%, were used for the source strength measurements.

After 10 years’ experience in conducting SMEQA, an on-site quality audit project was carried out from 2016 to 2019 to examine the SMEQA practice, assist clinical departments in improving quality in using medical exposure systems, and determine the development needs for regulation standards. This project was held by the medical physics society in Taiwan (CSMPT: Chinese Society of Medical Physics, Taipei), and the audit team consisted of six senior medical physicists and three well-trained auditors. The audit procedures were designed by senior medical physicists. The auditor performs the audit according to the instructions. All radiotherapy departments in Taiwan (81 in 2019) were included with yearly audit measuring the SMEQA core items including beam output, energy, uniformity, source strength, positioning et al. For about 141 linacs, 9 γ knife systems, 6 cyberknives, 20 Tomotherapys, and 34 HDR brachytherapy systems were audited yearly in this study.

Dosimetry audit can be performed by postal dosimeters, usually based on the thermoluminescent dosemeter (TLD) or optically stimulated luminescent dosemeter (OSLD) methods, for example as organized by the IAEA and the IROC-H. Another dosimetry audit method is on-site visits using ionization chambers and appropriate phantoms. On-site visits with directly acquired measurement readings have lower uncertainties, allow immediate feedback, discussion, and adjustment in a timely manner. Considering the hospital distribution is relatively concentrated in Taiwan, an on-site audit was adopted. The on-going dosimetry QA procedure and measurement devices performed by each department are tracked and checked using their own measurement tools. In this manner, the measurement data were acquired and the QA procedures, devices, and protocol parameters audited and validated.

## Methods and materials

### Data collection and analysis

Institutions were asked to provide documents including the QA operation procedure, ion chamber, and electrometer calibration reports from NRSL, reference dosimetry method and worksheet parameters for the calibration protocol for all medical therapy devices before performing on-site quality audit. In addition, a questionnaire relating to machine type, AEC licensing date for the therapy system, beam energy, MLC type, treatment planning system type, QA tools and staffing, etc., were recorded and sent back to the audit team.

The worksheet parameters for reference dosimetry for each beam provided by the institution were reviewed by the audit team. According to the reference-point dosimetry protocol used by the institute, discussion or revision request would be sent by the auditor if some parameters were inappropriately used. Prior to the audit measurements, the institution is asked to verify that the therapy unit is properly calibrated.

To track and check the on-going dosimetry QA procedure for each department, all QA devices and equipment used by the department for their QA measurements were provided to the auditors. All QA procedure and tools were reviewed and validated by the audit team before the on-site audit. According to government regulations, each of the ion chambers and electrometers used for the QA procedure must have a calibration certificate with issue date within 2 years by the NRSL. Parameter review and equipment validation can prevent potential errors in using the reference dosimetry protocol, and provide advice on improvement, where appropriate.

To perform an independent audit, an independent output measurement worksheet was created for each beam with the form designed by the audit team and independent parameters set according to the calibration protocol used by the institution.

### Quality audit procedure

The procedures and criteria audited include those listed in Table 1. The reference-point measurement for the linear accelerator and Cyberknife was performed using a 0.6 cc Farmer type ionization chamber and a homogeneous phantom. Tomotherapy and γ knife system use a specific ion chamber and solid phantom, such as Exradin A1SL (standard imaging, Middleton, Wisconsin) ion chamber and Virtual Water^™^ phantom (“cheese” phantom) for Tomotherapy, Semiflex (PTW-Freiburg, Germany) chamber and spherical polystyrene phantom for the γ knife to perform the reference-point measurement. Reference conditions for the determination of absorbed dose to water was checked on-site according to the reference dosimetry protocol used by the institution. Source strength measurement for brachytherapy system was made with well-type chamber, and the source(s) was placed centrally in the chamber at the most sensitive spot which has the same location as the calibration report done by NRSL. Central axis dosimetry parameter constancy, that is, beam quality check, was implemented by comparing the ${TPR}_{5}^{15}$ (or $TMR_{5}^{15}$ ) and the PDD_d≈80%PDD_ from baseline, respectively, for X-ray and electron beams. Beam flatness consistency and beam symmetry, *i.e.*that is, beam uniformity check, were implemented for both in-plane and cross-plane profiles at 10 cm depth and d_max_ for X-ray and electron beams, respectively, by the 80% of a 20 × 20 cm^2^ field for linac. Consistency with the baseline is specifically associated with flatness, and symmetry tolerance is regarded to the absolute value. The setting of the baseline value for beam quality check and beam uniformity check is according to the commissioning data from each institution. The output measurement chamber and commercialized two-dimensional dose measurement tool and the solid phantoms were usually used for monthly beam quality check and beam uniformity measurements, respectively. If these measurements were taken during the annual QA check, a water phantom system with beam profile scanning chamber will be used for these measurements. Consider the beam profiles for beams without flattening filters, including Tomotherapy and linac with flattening filter free (FFF) beams, beam profile constancy method stated in AAPM TG-142^10^ was used by comparing the QA measurements to the baseline off-axis factors at the same point locations that fall within 80% of an agreed upon field size.

**Table 1.** Procedures and acceptance criteria for on-site quality audits

Item	Procedure	Criterion
A.medical accelerator		
1	X-ray and electron output accuracy	2%
2	X-ray central axis dosimetry parameter consistency	2%
3	Electron central axis dosimetry parameter consistency	2% or 2 mm
4	X-ray beam flatness consistency	2%
5	Electron beam flatness consistency	3%
6	X-ray and electron beam symmetry	3%
B.HDR brachytherapy unit		
1	Source transit velocity consistency	1 sec (from shielded safe to total extension distance)
2	Source strength accuracy	5%
3	Source positioning accuracy	1 mm
4	Timer accuracy	1 sec/min
C.Tomotherapy		
1	X-ray output accuracy	2%
2	X-ray beam central axis dosimetry parameter consistency	2%
3	X-ray beam profile dosimetry parameter consistency	2%
4	Couch vertical/longitudinal motion accuracy	1 mm
D.Cyberknife		
1	X-ray output accuracy	2%
2	X-ray central axis dosimetry parameter consistency	2%
3	X-ray beam flatness consistency	3%
4	X-ray beam symmetry	3%
5	Imaging and treatment coordinate coincidence	1 mm
E.γ knife		
1	Photon output accuracy	2%
2	Coincidence of radiation and mechanical isocenter	0.3 mm
3	Timer accuracy	0.01 min

As γ knife and Cyberknife are specially designed for stereotactic radiosurgery or stereotactic radiotherapy, the QA item of the coincidence of radiation and mechanical isocenter was included in the audit measurement. As Tomotherapy delivers doses simultaneously with the gantry rotation and the treatment couch longitudinal motion, couch motion position accuracy for Tomotherapy was included.

EBT or RTQA radiochromic film was used for HDR system source position check. All audits refer to the QA operation procedure provided by the institution that has been validated by the AEC.

## Results

There were 141 linacs, 9 γ knife systems, 6 cyberknives, 20 Tomotherapys, and 34 HDR brachytherapy systems in Taiwan in 2019. External-beam radiotherapy (EBRT) treatment equipment was dominated by linacs with 80.1% of all EBRT treatment units being linacs. For linacs, most of photon beams audited (307 beams, 97.7%) were with energies of 6 MV (44.7%) or 10 MV (38.5%) or their FFF energy mode.

The radiation treatment resource is about 8.9 therapy machines or 7.5 therapy machines with MV/MeV beams per million population in Taiwan. About 1.20 medical physicists are available per radiation therapy unit. All audit measurements in this study were within the SMEQA acceptance criteria.

### Reference point and beam quality measurements

AAPM TG-21 is the most used reference dosimetry protocol for linac output calibration (67.4%). However, for increasingly more new installed linacs have FFF beams, because the addendum report to the AAPM’s TG-51 protocol^17^ considers applicability to FFF beams, so the facilities that use the TG-51 protocol as the basis for dose calibration are gradually increasing. Additionally, as the IAEA TRS-398 is the only present code on absorbed dose standards to water for proton and heavy-ion beams, therefore, several facilities that have installed or intend to install particle therapy systems are changing the protocol for using TRS-398 as the dose calibration protocol to facilitate calibration system integration.

**Figure 1.**
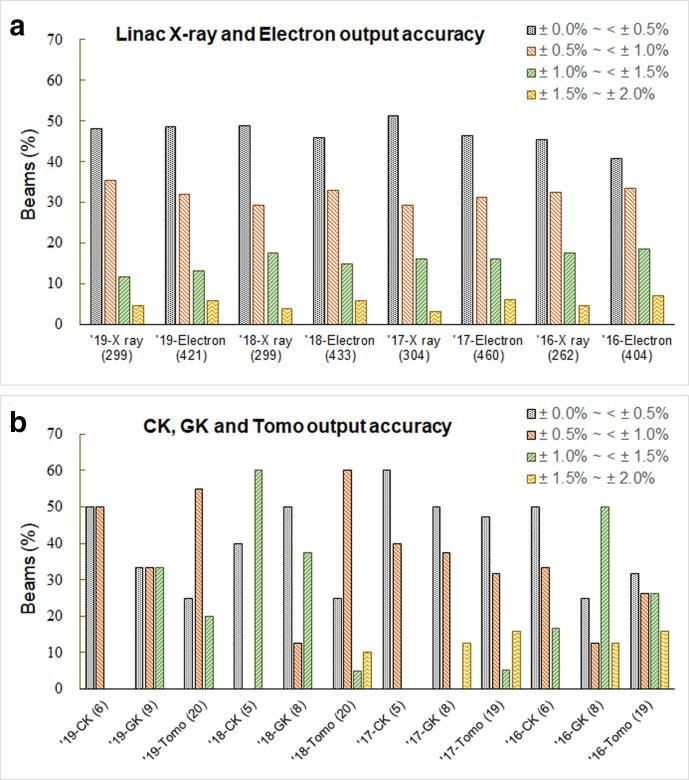
Results for the reference-point measurement showing the deviation distribution of the measured dose ratio to the stated dose. (a) photon and electron beams from linacs. (b) measurements from γ knife systems, Cyberknives, and Tomotherapys.

**Figure 2.**
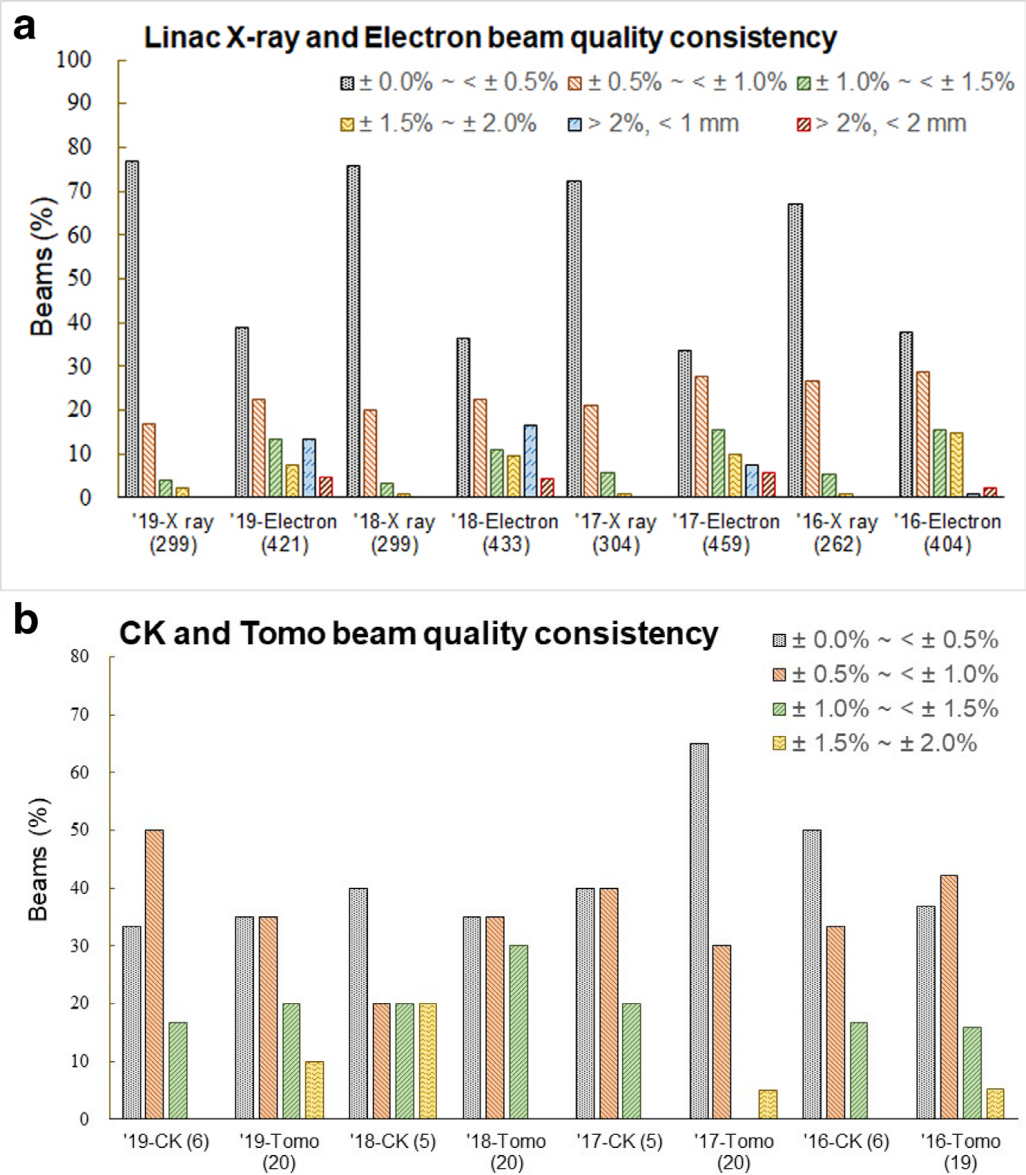
Results for the beam quality measurement. (a) photon and electron beams from linacs. (b) measurements from γ knife systems, Cyberknives, and Tomotherapys.

More than 300 photon beams from linacs, γ knife systems, Cyberknives, and Tomotherapys, and more than 400 electron beams from linacs each year were measured and the results are shown in Figures 1 and 2. According to the SMEQA criterion, the deviation classification of audit measurement results is at 0.5% intervals. For reference-point measurements, more than 78 and 75% of the photon beams and electron beams respectively from linacs were with measured dose deviations within ±1.0% (Figure 1a). The beam control system of the Cyberknife is similar to that of a linear accelerator. The reference-point dose deviation is also similar to that of a linac system. (Figure 1b). The linac has shown a more stable output distribution than the Tomotherapy system.

For beam quality measurements, photon beams have smaller deviations than electron beams. More than 90% of the photon beams from linacs were with measured beam quality deviation within ±1.0% (Figure 2a). Although the depth dose values of the electron beams have a larger deviations at the set depth than the photon beams, but all the depth deviations were within 2 mm.

### Beam uniformity

**Figure 3.**
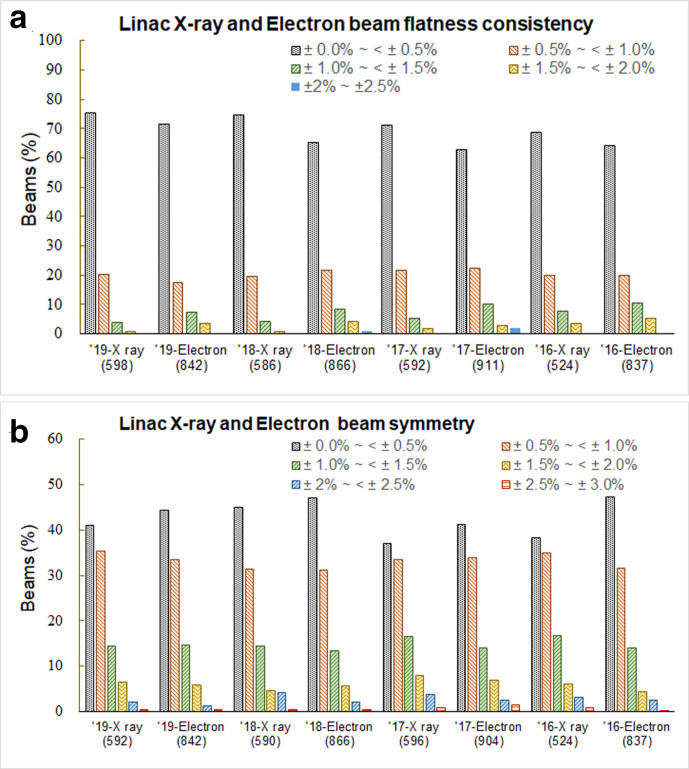
Results for the Beam uniformity measurement for photon and electron beams from linacs. (a) flatness consistency; (b) symmetry.

Including in-plane and cross-plane, about 600 photon and 850 electron profiles from linacs were measured each year. More than 90 and 85% of photon and electron measurements, respectively, with flatness consistency ≤±1.0% (Figure 3a). Beam symmetry as an absolute value, regardless of baseline, have more than 75% of measurements ≤±1.0% (Figure 3b).

### HDR brachytherapy QA measurements

The measured reference air kerma rate deviation to the vendor’s certificate was determined. For 117 measurements in the study period, most of the deviations were within ±3%, except in one case, which was −3.2%, being still within the ±5% source calibration uncertainty quoted by the vendors. All measurements of source transit velocity consistency were within 1 sec, and the timer accuracy were within 1 sec/min. The source positioning accuracy was measured using more than two source dwell positions (±30 mm), and all deviations were within 1 mm.

### Mechanical motion accuracy

All couch motion deviations for Tomotherapy, Cyberknife, and γ knife were within 1 mm, and the radiation isocenter deviations relative to the mechanical isocenter and imaging isocenter for γ knife systems and Cyberknives were within 0.3 mm and 1 mm, respectively.

## Discussion

According to IAEA DAN data, in the countries that provide dosimetry audit services for radiotherapy, more than a quarter are due to government regulations.^7^ Although the government regulation exists in Taiwan, the lack of framework to provide routine dosimetry audit, an on-site measurement audit supported by governmental agency was implemented for all radiation therapy units from 2016 to 2019. The core items in the SMEQA were included.

A comprehensive quality assurance should cover the whole radiotherapy process, such as patient positioning, treatment planning, patient-specific dosimetry measurements, and machine performance checks. However, routine machine QA is fundamental to assure and maintain that the machine characteristics do not deviate significantly from their baseline values set at the time of acceptance and commissioning, and to fulfill the needs for dosimetry accuracy.

In order to perform an independent audit, the audit team should have independent dose measurement tools and dose calculation methods. This study uses their own measurement tools provided by the auditee, which makes this study have the drawbacks of lack of independence. Considering that this procedure may cause uncertainty and potential lack of comparability due to the use of different measurement tools, the audit team strictly requires the auditee to provide a complete calibration certificate for each dose measurement tool. Only after confirming that the auditee department has used the measurement devices and calibration methods correctly, and the audit team has created an independent output measurement worksheet, the on-site audit will be conducted. The on-site audit was carried out in the company of the medical physicist of the auditee department. All procedures must follow AEC-certified operating procedures, and the measuring equipment used must also be consistent with the previously provided data. In this way, the effectiveness and accuracy of the equipment used in each audit can be guaranteed, and the correctness of the equipment and methods used by the auditee can be confirmed on-site.

In addition to measurement audits, according to Taiwan’s “Ionizing Radiation Protection Act”, each radiotherapy device needs to be inspected by AEC officials for radiation safety before it is approved for clinical use. Inspections include dose rate measurement in the work environment, as well as document review of the commissioning of the treatment equipment, including the treatment machine and treatment planning system (TPS). Under this procedure, the accuracy of the calculated dose by TPS can be initially confirmed.

### Treatment resource and medical physicist staffing

The treatment resource in Taiwan is about 7.5 therapy machines with MV/MeV beams per million populations. This is roughly similar to Australia and France.^18,19^ According to the IAEA radiotherapy practice survey,^20^ the staffing levels for those departments without dosimetrists there were on average 1.3 medical physicists per treatment unit, and this level is at the low end range compared with IAEA recommendations.^21^ From this study, there were on average 1.2 medical physicists per treatment unit in Taiwan, this level is lower than the worldwide average, although the treatment resources in Taiwan are much greater than the worldwide average. This survey reflects that radiation oncology medical physicists in Taiwan generally are overworked, just like the reports from IAEA worldwide survey and the Asia Pacific region survey.^19,20^

### Reference point and beam quality measurements

Most of the measured-to-stated doses ratios for the reference-point measurements were within ±1.0% (Figure 1), showing that no major systematic errors exist. All reference-point values lay within ±2% of unity. The mean differences and standard deviations (SD) in 2017 from this study were 0.03 and 0.68% for linac photon beams, and were 0.01 and 0.75% for electron beams, respectively. These results are similar to the reference dosimetry audit report in the UK since 2003 that with the mean differences and SDs of 0.3 and 0.4% for MV photon beams, 0.3 and 0.7% for electron beams.^3^ These results are better than the 2016–2018 results from the IAEA/WHO postal dose audit for low-income and middle-income countries worldwide.^22^ From Figure 2, photon beams have shown smaller variations in beam energy than electron beams. For electron beams, different depth combinations were selected for checking beam quality, and the selected depth of about 80% PDD may be larger fall-off gradient than photon beams. This makes higher measurement uncertainty for electron beams than photon beams. However, all deviations in depth were within 2 mm.

Homogeneous solid phantoms were used in this measurement. Different solid phantoms were used, and more than 50% institutions in Taiwan using RW3 white water (PTW-Freiburg, Germany) for dosimetry measurements. Tello’s study showed that solid phantom materials could cause a range of 3–4% in the dose relative to the dose determined from measurements in water.^23^ Attention should be paid before any solid phantom material is used as a water substitute. A solid-phantom-to-water correction factor was asked to apply during the document review process if this phantom material is not used in the reference dosimetry protocol. The method of using the ion chamber measurement ratios between solid phantoms and water to determine the phantom-to-water conversion factor was suggested to the department’s medical physicists by the audit team.^24^

In addition to SMEQA system establishment and education and training implementation, the machine quality is also closely related to machine maintenance implementation. Taiwan is a densely populated area, and the resource spatial distribution is also dense. Vendor maintenance teams are able to arrive on site within a short time, to repair machine malfunctions or perform routine maintenance services. Even for QA-related adjustments, medical physicists and engineers usually work together to complete the QA procedure and parameter adjustments if needed for achieving the goal of maintaining high quality machine performance.

## Conclusions

On-site machine QA audits were implemented from 2016 to 2019 for all radiation therapy units each year. These measurement results have shown high-quality machine performance in Taiwan. Accompanying the SMEQA audits, surveys for small field dosimetry, and IMRT and VMAT plan calculation accuracy, and image guided system quality surveys also have been performed. The data is in processing and would soon be published.
